# The association between anthropometric measures of adiposity and the progression of carotid atherosclerosis

**DOI:** 10.1186/s12872-020-01417-0

**Published:** 2020-03-17

**Authors:** Yume Imahori, Ellisiv B. Mathiesen, Katy E. Morgan, Chris Frost, Alun D. Hughes, Laila A. Hopstock, Stein Harald Johnsen, Nina Emaus, David A. Leon

**Affiliations:** 1 Department of Non-communicable Disease Epidemiology, Faculty of Epidemiology and Population Health, London School of Hygiene & Tropical Medicine, Keppel Street, London, WC1E 7HT UK; 2 Department of Clinical Medicine, UiT The Arctic University of Norway, Tromsø and Department of Neurology, University Hospital of North Norway, 9037 Tromsø, Norway; 3 Department of Medical Statistics, London School of Hygiene & Tropical Medicine, London, WC1E 7HT UK; 4 Department of Population Science & Experimental Medicine, & MRC Unit for Lifelong Health and Ageing, University College London, London, WC1E 6BT UK; 5 Department of Community Medicine, Faculty of Health Sciences, UiT The Arctic University of Norway, 9037 Tromsø, Norway; 6 Department of Health and Care Sciences, Faculty of Health Sciences, UiT The Arctic University of Norway, 9037 Tromsø, Norway

**Keywords:** Obesity, Abdominal obesity, Atherosclerosis, Carotid plaque, Epidemiology

## Abstract

**Background:**

Few reports are available on the contribution of general and abdominal obesity to the progression of carotid atherosclerosis in late adulthood. This study investigated the impact of four simple anthropometric measures of general and abdominal obesity on the progression of carotid atherosclerosis and the extent to which the association between adiposity and the progression of plaque burden is mediated by cardiometabolic markers.

**Methods:**

Four thousand three hundred forty-five adults (median age 60) from the population-based Tromsø Study were followed over 7 years from the first carotid ultrasound screening to the next. The progression of carotid atherosclerosis was measured in three ways: incidence of plaques in previously plaque-free participants; change in the number of plaques; and total plaque area (TPA). We used generalised linear models to investigate the association between each adiposity measure – body mass index (BMI), waist circumference (WC), waist-to-hip ratio (WHR), and waist-to-height ratio (WHtR) – and each outcome. Models were adjusted for potential confounders (age, sex, smoking, education, physical activity). The pathways through which any associations observed might operate were investigated by further adjusting for cardiometabolic mediators (systolic blood pressure, cholesterol, and HbA1c**).**

**Results:**

There was little evidence that adiposity was related to the formation of new plaques during follow-up. However, abdominal adiposity was associated with TPA progression. WHtR showed the largest effect size (mean change in TPA per one standard deviation (SD) increase in WHtR of 0.665 mm^2^, 95% confidence interval 0.198, 1.133) while BMI showed the smallest. Effect sizes were substantially reduced after the adjustment for potential mediators.

**Conclusions:**

Abdominal obesity indirectly measured with WC seems more strongly associated with the progression of TPA than general obesity. These associations appear to be largely mediated by known cardiometabolic markers.

## Background

Obesity is a global epidemic. In 2016, 39% of adults worldwide were estimated to be overweight, and 13% obese [1]. The global prevalence of obesity has almost tripled over the last 30 years and continues to increase [1]. This trend is of substantial concern, as obesity is associated with an increase in cardiometabolic disorders and cardiovascular disease (CVD), the leading cause of death worldwide. Paradoxically, over this period, the burden of CVDs has been decreasing in industrialised countries [2, 3]. However, the current increase in obesity could offset or reverse the downward trend of CVD mortality in spite of the decrease in the prevalence of other traditional CVD risk factors, such as smoking and hypertension [3]. A recent analysis of US data has suggested that if body mass index (BMI) had not increased, life expectancy in 2011 at age 40 would be 0.9 years higher than was actually the case [4], which in part would be due to a slowdown in the rate of decline of CVD mortality [5].

Atherosclerosis is a major cause of CVD. Carotid atherosclerosis is easily and non-invasively detected using ultrasound. Carotid intima-media thickness (IMT) consistently predicts future CVD events, but carotid plaque outperforms IMT regarding its predictive ability of future CVD [6]. Most previous studies on the association between obesity and carotid atherosclerosis in adults have been cross-sectional. Relatively few studies have attempted to look at this relationship prospectively. One population study investigated the association between lifetime BMI and IMT in late adulthood and showed that a reduction in the BMI category was associated with a decrease in IMT [7]. Several others have investigated determinants of carotid plaque progression, including obesity as one of the potential factors [8–11].

If there is evidence of obesity influencing progression, this is of direct relevance to clinical management in adulthood. Also, compared to general obesity (the exposure in most previous studies), abdominal obesity might play a more important role in progression because of its stronger association with cardiometabolic diseases [12].

In an earlier investigation of cross-sectional data from the population-based Tromsø Study, we have shown that abdominal obesity was more closely associated with carotid plaque burden assessed by total plaque area (TPA) than general obesity [13]. We also found that cardiometabolic risk factors such as hyperlipidemia, glucose intolerance, and hypertension mediated much of this association. The aim of the current analysis is to extend these investigations to determine whether the progression of carotid plaque burden over 7 years is related to different measures of obesity in the Tromsø Study, and how far any indication of such an association is mediated by the same set of cardiometabolic risk factors.

## Method

### Study design and participants

The Tromsø Study [14] is an ongoing population-based prospective cohort study based on the population of the municipality of Tromsø in Northern Norway; it consists of seven surveys spanning 1974–2016 (Tromsø 1–7).

#### Baseline (1994–95)

The Tromsø Study’s fourth survey (the 4th survey), conducted in 1994–95, is the largest survey to date, inviting everyone aged 25 years and above living in the Tromsø municipality. Among the 4th survey’s participants, all those aged 55–74 years, plus sampling fractions between 5 and 10% from other age groups, were eligible for a second visit with extensive clinical examinations including carotid ultrasound, with 76% (*n* = 6727) attending [15]. All of the 4th survey’s second visit participants were invited to take part in the 5th (2001) and 6th (2007–08) surveys for a follow-up ultrasound examination.

#### Seven-year follow-up at the 5th survey (2001)

All of the 4th survey’s second visit participants, except those who had died (*n* = 499) or moved from Tromsø (*n* = 372), were invited to the 5th survey’s ultrasound examination (*n* = 5856), with 83% attending (*n* = 4858), i.e. 72% of the participants from the 4th survey’s second visit were rescanned. After excluding participants without valid written consent (*n* = 29) and with missing data on the main covariates (*n* = 484), 4345 participants were included in the current analysis of the seven-year follow-up (1994–2001) data.

#### Fourteen-year follow-up at the 6th survey (2007–08)

All of the 4th survey’s second visit participants, except those who had died (*n* = 1515) or moved from Tromsø (*n* = 468), were also invited to the 6th survey’s ultrasound examination (*n* = 4744), with 63% attending (*n* = 2975), i.e. 44% of the participants from the 4th survey were rescanned. After excluding those who withdrew their consent (n = 1) or had missing covariates (*n* = 289), 2685 participants with complete repeated measurements were eligible for the current analysis of 14-year follow-up (1994–2008) data.

The Regional Committee for Research Ethics and the Norwegian Data Inspectorate have approved the Tromsø Study study. All participants included in the present study have given written informed consent.

### Measurement of obesity and other CVD risk factors: the baseline 4th survey (1994–95)

Height and weight were measured with light clothing and without shoes using standard methods. Waist circumference (WC) was measured at the level of the umbilicus while hip circumference was measured at the widest part of the thigh to the nearest 0.5 cm. BMI was calculated by dividing body weight by height squared. Waist-to-hip ratio (WHR) and waist-to-height ratio (WHtR) were calculated by dividing WC by hip circumference and height, respectively. Categorical adiposity variables were created with three BMI levels (BMI < 25 kg/m^2^, 25 < =BMI < 30 kg/m^2^ (overweight), BMI > =30 kg/m^2^ (obesity)), two WC levels (abdominal obesity: men> 102 cm, women> 88 cm), and two WHR levels (abdominal obesity: men> = 0.9, women> = 0.85), according to WHO cut-off points [16].

Information on current smoking (yes/no), education level (primary education, vocational/high school, university/college), physical activity (hours spent on leisure physical activity (sweating/out of breath) per week in leisure time: none, less than one, one to two, three or more), and medical history (myocardial infarction, angina pectoris, stroke, diabetes) was obtained by self-administered questionnaires. Non-fasting serum total cholesterol, triglycerides, and high-density lipoprotein cholesterol (HDL-C) were determined using standardised methods. Low-density lipoprotein cholesterol (LDL-C) was calculated using the Friedewald equation. HbA1c was measured using the Cobas Mira instrument. Blood pressure was measured seated three times with a one-minute interval between readings after one minute of seated rest, using an automatic device (Dinamap Vital Signs Monitor 1846; Critikon Inc., Tampa, FL, USA). The average of the last two blood pressure measurements was used in the analysis.

### Ultrasound examination: the 4th, 5th, and 6th surveys (1994–2008)

The right carotid artery was scanned in the far and near wall of the common carotid artery, bifurcation, and internal carotid artery to seek for carotid plaques using an Acuson Xp 10,128 ART ultrasound scanner equipped with a 7.5 MHz linear-array transducer in the 4th and 5th surveys, and a GE Vivid 7 scanner with a linear 12 MHz transducer in the 6th survey, as previously described [17, 18]. The same protocol was used in all surveys. Carotid plaque was defined as a focal structure encroaching into the arterial lumen at least 0.5 mm or 50% of the surrounding IMT value or IMT > 1.5 mm as measured from the media-adventitia interface to the intima-lumen interface. Cine loops and still images of each carotid plaque in the longitudinal plane were stored and digitalised for offline analysis. All plaque images were outlined manually to assess plaque areas using the Adobe Photoshop imaging-procession programme (ver. 7.0.1). When there were multiple plaques, all plaque areas were summarised to calculate TPA. Inter-equipment variability and intra-reader and inter-reader reproducibility of plaque detection and measurement of plaque area were acceptable [17, 18].

### Statistical analysis

#### Primary analysis: changes from the baseline 4th survey to the 5th survey (1994–2001)

Baseline characteristics were summarised as means with standard deviations (SD) (or medians with the inter-quartile range if markedly non-normally distributed) for continuous variables, and counts and frequencies (percentages) for categorical variables.

We used three outcomes to assess the progression of carotid atherosclerosis: new plaque formation (binary yes/no) in those without plaque at baseline; change in the number of plaques from baseline to follow-up; and change in TPA over the same period.

To directly compare the strength of association of each adiposity measure, taking account of the different distributions in men and women, we calculated sex-specific standardised adiposity scores by subtracting the sex-specific mean of each adiposity measure from the observed value and then dividing by the sex-specific SD. The association between new plaque formation and each adiposity measure was examined using a series of logistic regression models. The effect of each adiposity measure on the change in the number of plaques/TPA was estimated using analogous linear regression models. Due to concerns over the normality assumption, we also constructed confidence intervals (CIs) using a bootstrap approach, as described in the Appendix. Separately for each outcome variable and each adiposity measure, we fitted a sequence of three models. We selected potential confounders and mediators a priori based on evidence of the association between obesity and CVD. Model 1 was adjusted for age (categorical five-year interval) and sex. Model 2 was further adjusted for the other potential behavioural and socio-demographic confounders (smoking, physical activity, education), this being our main model for estimating the association between adiposity and the progression of atherosclerosis. To assess the extent to which any observed associations in model 2 might be mediated by cardiometabolic risk factors, model 3 was further adjusted for the following potential mediators: systolic blood pressure, HDL-C, LDL-C, and glycated haemoglobin as continuous variables, and antihypertensive- and lipid-lowering drug use and existing diabetes as binary variables.

STATA version 14 (StataCorp) was used for all analyses.

#### Secondary analyses: changes from the baseline 4th survey to the 6th survey (1994–2008)

We conducted parallel analyses to those above using the 6th survey follow-up data (2008). In planning this study, we had intended to focus equally on this analysis. However, having undertaken the analysis, the sample size was appreciably smaller after the 14-year follow-up, and effect estimates markedly less precise than those from our 5th survey analysis. Therefore, we focus our presentation on the 5th survey analysis. As our initial intention was to include analyses from the 6th survey data and to avoid publication bias, analyses involving the 6th survey after the 14-year follow-up are reported only in supplementary tables, although the results were largely uninformative.

## Results

### Baseline characteristics

Table 1 shows participant characteristics at baseline. The median age of participants was 60 years (interquartile range 55–66), and 51% were women. Compared to the 4th survey’s second visit participants who did not attend the 5th or 6th surveys, the attending population was younger and with a more favourable CVD risk profile (Tables S1–S2). Adiposity measures were approximately normally distributed. According to the BMI classification, 44.4 and 13.4% of the participants were categorised as overweight and obesity, respectively. Increased WC (men> 102 cm, women> 88 cm) was observed in 28.3% of the participants while 42.7% had increased WHR (men> = 0.9, women> = 0.85). Lipid-lowering drug use prevalence was 2.6%.

**Table 1:** Participant characteristics at baseline (Tromsø Study the 4th survey: analysis restricted to participants with all covariates and outcomes at both 4th and 5th survey: *n* = 4345)

	Total (4345)	Men (2114)	Women (2231)
Age (years) median (IQR)	60 (55–66)	60 (55–65)	61 (56–67)
*Anthropometric measures Mean ± SD*			
Height (cm)	168.5 ± 9.4	175.6 ± 6.7	161.8 ± 6.2
Weight (kg)	74.0 ± 13.0	80.6 ± 11.5	67.8 ± 11.2
BMI (kg/m^2^)	26.0 ± 3.7	26.1 ± 3.2	25.9 ± 4.2
WC (cm)	89.7 ± 11.0	95.0 ± 9.0	84.7 ± 10.5
WHR	0.87 ± 0.08	0.92 ± 0.06	0.82 ± 0.06
WHtR	0.53 ± 0.06	0.54 ± 0.05	0.52 ± 0.07
*Categorical obesity*^*a*^*N(%)*			
BMI < 25 kg/m^2^	1836 (42.3)	803 (38.0)	1033 (46.3)
25 < =BMI < 30 kg/m^2^ (Overweight)	1930 (44.4)	1087 (51.4)	843 (37.8)
BMI > =30 kg/m^2^(Obesity)	579 (13.3)	224 (10.6)	355 (15.9)
WC: m > 102 cm, w > 88 cm (Abdominal obesity)	1226 (28.2)	449 (21.2)	777 (34.8)
WHR m > =0.9 f > =0.85(Abdominal obesity)	1850 (42.6)	1245 (58.9)	605 (27.1)
*Potential confounders N(%)*			
Current Smoker	1290 (29.7)	657 (31.1)	633 (28.4)
Physical activity (hours/week) ^b^			
None	2666 (61.4)	1077 (51.0)	1589 (71.2)
Less than 1	673 (15.5)	381 (18.0)	292 (13.1)
1–2	670 (15.4)	421 (19.9)	249 (11.2)
3 or more	336 (7.7)	235 (11.1)	101 (4.5)
Education			
Primary education	2229 (51.3)	910 (43.1)	1319 (59.1)
Vocational/high school	1328 (30.6)	738 (34.9)	590 (26.5)
University/college	788 (18.1)	466 (22.0)	322 (14.4)
*Potential mediators Mean ± SD*			
Systolic blood pressure (mmHg)	143.4 ± 21.5	143.6 ± 19.4	143.2 ± 23.2
Total cholesterol (mmol/l)	6.69 ± 1.27	6.51 ± 1.18	6.85 ± 1.32
Triglycerides (mmol/l)	1.51 ± 0.88	1.62 ± 0.96	1.41 ± 0.79
HDL cholesterol (mmol/l)	1.52 ± 0.44	1.37 ± 0.39	1.66 ± 0.44
LDL cholesterol (mmol/l)	4.48 ± 1.17	4.40 ± 1.09	4.55 ± 1.22
HbA1c (%)	5.44 ± 0.63	5.42 ± 0.57	5.47 ± 0.68
*Medical history/Medication N(%)*			
Myocardial infarction	197 (4.5)	156 (7.4)	41 (1.8)
Angina pectoris	335 (7.7)	198 (9.4)	137 (6.1)
Stroke	83 (1.9)	42 (2.0)	41 (1.8)
Diabetes	96 (2.2)	46 (2.2)	50 (2.2)
Lipid-lowering drug use	114 (2.6)	64 (3.0)	50 (2.2)
Blood pressure lowering drug use	515 (11.9)	248 (11.7)	267 (12.0)
*TPA at T4 (IQR)*			
TPA Q1	0–0	0–0	0–0
TPA Q2	0–0	0–4.0	0–0
TPA Q3	0–12.9	4.0–16.2	0–10.4
TPA Q4	12.9–135.1	16.2–135.1	10.4–121.2
*Number of plaques at T4 N (%)*			
0	2337 (53.8)	1027 (48.6)	1310 (58.7)
1	1268 (29.2)	655 (31.0)	613 (27.5)
2	534 (12.3)	295 (14.0)	239 (10.7)
3	147 (3.4)	95 (4.5)	52 (2.3)
4	49 (1.1)	35 (1.7)	14 (0.6)
5	9 (0.2)	7 (0.3)	2 (0.1)
6	1 (0.02)		1 (0.04)

*BMI* body mass index, *WC* waist circumference, *WHR* waist-to-hip ratio, *WHtR* waist-to-height ratio, *HDL* high-density lipoprotein, *LDL* low-density lipoprotein, *HbA1c* glycated haemoglobin, *TPA* total plaque area

^a^Increased waist circumference (> 102 cm for men, > 88 cm for women) and increased WHR (> = 0.9 for men, > = 0.85 for women) are categorised according to WHO cut-off points [1]. ^b^Hours spent on hard physical activity (sweating/out of breath) in leisure time

At baseline, 46.2% of participants had at least one plaque. The median TPA in subjects with plaques was 15.7 mm^2^ in men and 12.7 mm^2^ in women.

### Carotid plaque burden at seven-year follow-up

Table 2 shows carotid plaque burden at the 5th survey by category of adiposity 7 years earlier at the 4th survey. At the seven-year follow-up, 62.2% of the participants had at least one plaque. Among the 2337 participants without plaque at the baseline 4th survey, 40% developed at least one new plaque. Median TPA increased from 0 mm^2^ at baseline to 8.9 mm^2^. Participants who belong to a higher adiposity category were more likely to have a higher plaque burden and greater progression of plaque burden compared to those in lower categories.

**Table 2:** Carotid plaque at the 5th survey (2001) according to adiposity category at the baseline 4th survey (1994–95) (n = 4345)

	Total population	BMI categories			WC categories_a_		WHR categories_a_	
Carotid variable	n/N (%)	BMI < 25 kg/m^2^ n/N (%)	25 < =BMI < 30 kg/m^2^ (Overweight) n/N (%)	BMI > =30 kg/m^2^ (Obesity) n/N (%)	Abdominal obesity		Abdominal obesity	
					No n/N (%)	Yes n/N (%)	No n/N (%)	Yes n/N (%)
At least one plaque at T5	2704/4345 (62.2)	1078/1836 (58.7)	1250/1930 (64.8)	376/579 (64.9)	1901/3119 (61.0)	803/1226 (65.5)	1414/2495 (56.7)	1290/1850 (69.7)
New plaques at T5_b_	938/2337 (40.1)	374/1021 (36.6)	450/1028 (43.8)	114/288 (39.6)	660/1703 (38.8)	278/634 (43.9)	521/1454 (35.8)	417/883 (47.2)
Number of plaques at T5								
0	1641 (37.8)	758 (41.3)	680 (35.2)	203 (35.1)	1218 (39.1)	423 (34.5)	1081 (43.3)	560 (30.3)
1	1230 (28.3)	478 (26.0)	578 (30.0)	174 (30.1)	861 (27.6)	369 (30.1)	675 (27.1)	555 (30.0)
2	869 (20.0)	361 (19.7)	378 (19.6)	130 (22.5)	614 (19.7)	255 (20.8)	463 (18.6)	406 (22.0)
+ 3	605 (13.9)	239 (13.0)	294 (15.2)	72 (12.4)	426 (13.7)	179 (14.6)	276 (11.1)	329 (17.8)
Change in number of plaques from T4 to T5								
Decreased	442 (10.2)	181 (9.9)	198 (10.3)	63 (10.9)	304 (9.8)	138 (11.3)	243 (9.7)	199 (10.8)
No change	2191 (50.4)	960 (52.3)	943 (48.9)	288 (49.7)	1612 (51.7)	579 (47.2)	1349 (54.1)	842 (45.5)
Increased	1712 (39.4)	695 (37.9)	789 (40.9)	228 (39.4)	1203 (38.6)	509 (41.5)	903 (36.2)	809 (43.7)
	Median (IQR)							
TPA at T5 (mm^2^)	8.9 (1–22.6)	7.5 (0–20.7)	9.7 (0–24.6)	10.9 (0–24.3)	8.3 (0–21.7)	10.4 (0–24.4)	6.6 (0–18.8)	12.5 (0–28.8)
Change in TPA from T4 to T5 (mm^2^)	0 (0–12.1)	0 (0–10.6)	1.6 (0–13.1)	0.7 (0–12.1)	0 (0–11.5)	1.7 (0–13.5)	0 (0–9.3)	3.7 (0–15.2)

Data are count and percentage for binary plaque and median and IQR for number of plaques and TPA. BMI: body mass index, T4: Tromsø Study the fourth survey, T5: Tromsø Study the fifth survey, TPA: total plaque area

WC: waist circumference, WHR: waist-to-hip ratio, WHtR: waist-to-height ratio, TPA: total plaque area

^a^Abdominal obesity WC: > 102 cm for men, > 88 cm for women, WHR: > = 0.9 for men, > = 0.85 for women (categorised according to WHO cut-off points, _b_ New plaques at T5: The number of participants who developed at least one plaque among participants without plaque at T4

### New plaque formation among participants without plaque at baseline (baseline – the 5th survey)

Table 3 shows odds ratios for developing at least one plaque at the seven-year follow up among the 2337 participants without plaque at baseline per 1 SD increase in each adiposity index. After adjustment for confounders only, all estimated odds ratios were small and non-significant.

**Table 3:** Odds ratios of having plaques at the 5th survey (2001) among participants without plaques at the baseline 4th survey (1994–95), by adiposity at the 4th survey (incident plaque when plaque is absent at the 4th survey) (*n* = 2337)

	Model 1 OR (95%CI)	*p*-value	Model 2 OR (95%CI)	*p*-value	Model 3 OR (95%CI)	*p*-value
BMI (per 1 SD)	0.98 (0.89, 1.08)	0.75	1.03 (0.93, 1.13)	0.60	0.94 (0.85, 1.05)	0.29
WC (per 1 SD)	1.01 (0.92, 1.11)	0.87	1.04 (0.94, 1.15)	0.44	0.96 (0.86, 1.07)	0.49
WHR (per 1 SD)	1.06 (0.96, 1.16)	0.24	1.06 (0.97, 1.17)	0.20	1.01 (0.92, 1.12)	0.77
WHtR (per 1 SD)	1.04 (0.94, 1.14)	0.49	1.07 (0.96, 1.18)	0.21	0.98 (0.87, 1.09)	0.68

*BMI* body mass index, *WC* waist circumference, *WHR* waist-to-hip ratio, *WHtR* waist-to-height ratio, *OR* odds ratio, *95% CI* 95% confidence interval, *SD* standard deviation, Model 1: adjusted for age and sex, Model 2: adjust for variables in Model 1 plus other confounders (smoking, physical activity and education), Model 3: adjusted for variables in Model 2 and mediators (systolic blood pressure, HDL cholesterol, LDL cholesterol, glycated haemoglobin, diabetes, lipid and blood pressure-lowering drug)

### Progression of plaque number (baseline – the 5th survey)

Table 4 shows the estimated changes in the number of plaques per 1 SD increase in each adiposity measure at baseline. After adjustment for confounders (model 2), no adiposity measure except for WHR was significantly associated with changes in the number of plaques.

**Table 4:** Changes in the number of plaques and total plaque area between the baseline 4th survey (1994–95) and the 5th survey (2001) per 1 SD increase in baseline adiposity (number of plaques: n = 4345, total plaque area: *n* = 4302)

**Number of plaques**						
	Model 1 β (95%CI)	*p*-value	Model 2 β (95%CI)	*p*-value	Model 3 β (95%CI)	*p*-value
BMI (per 1 SD)	−0.016 (−0.047, 0.015)	0.32	0.004 (− 0.028, 0.035)	0.82	− 0.011 (− 0.046, 0.023)	0.51
WC (per 1SD)	0.001 (− 0.030, 0.032)	0.97	0.014 (− 0.017, 0.045)	0.38	0.002 (− 0.031, 0.036)	0.89
WHR (per 1 SD)	0.029 (−0.002, 0.060)	0.07	0.032 (0.001, 0.064)	0.04	0.028 (−0.005, 0.061)	0.09
WHtR (per 1 SD)	0.010 (−0.021, 0.042)	0.52	0.024 (−0.008, 0.056)	0.14	0.013 (−0.021, 0.048)	0.45
**Total plaque area**						
BMI (per 1 SD)	0.184 (−0.298, 0.666)	0.45	0.412 (−0.077, 0.902)	0.10	0.005 (−0.525, 0.534)	0.99
WC (per 1 SD)	0.468 (−0.014, 0.949)	0.06	0.608 (0.122, 1.094)	0.01	0.261 (−0.260, 0.782)	0.33
WHR (per 1 SD)	0.698 (0.211, 1.185)	0.005	0.697 (0.209, 1.186)	0.005	0.471 (−0.038, 0.980)	0.07
WHtR (per 1 SD)	0.614 (0.122, 1.106)	0.01	0.750 (0.252, 1.249)	0.003	0.397 (−0.138, 0.932)	0.15

*BMI* body mass index, *WC* waist circumference, *WHR* waist-to-hip ratio, *WHtR* waist-to-height ratio, *OR* odds ratio, *95% CI* 95% confidence interval, *SD* standard deviation, Model 1: adjusted for age and sex, Model 2: adjust for variables in Model 1 plus other confounders (smoking, physical activity and education), Model 3: adjusted for variables in Model 2 and mediators (systolic blood pressure, HDL cholesterol, non-HDL cholesterol, glycated haemoglobin, diabetes, lipid and blood pressure-lowering drug)

### Progression of TPA (baseline – the 5th survey)

Table 4 shows the estimated changes in TPA per 1 SD increase in each adiposity measure at baseline. After adjustment for confounders (model 2), there were statistically significant associations between the progression in TPA and all adiposity measures except for BMI. WHtR showed the largest regression coefficient, followed by WHR, and BMI showed the smallest. After further adjustment for potential mediators (model 3), all effect sizes decreased substantially, and all became non-significant.

### Fourteen-year follow-up (baseline - the 6th survey)

Analogous tables to those above for the 6th survey follow-up data are shown in Tables S3–5. These analyses included markedly fewer people than those for the 5th survey follow-up, and effect estimates were consequently considerably less precise with wide CIs.

## Discussion

In this seven-year follow-up of a population-based sample of women and men in late adulthood, we found that abdominal obesity was more strongly associated with the progression of carotid plaque burden than general obesity. New plaque formation among participants without plaque at baseline was, however, not associated with any adiposity measures. Furthermore, all significant associations observed were in part mediated by cardiometabolic risk factors.

Our results suggest that abdominal obesity in late adulthood might contribute to the progression of carotid plaque burden with larger effect estimates than general obesity, imposing an excess risk on the progression of atherosclerosis. This finding concurs with our previous findings from a cross-sectional analysis, although considerable overlap of CIs prevents us from drawing definitive conclusions [13].

Several previous studies have investigated the effect of general obesity on the progression of carotid atherosclerosis [8–10]. Herder et al. investigated the determinants of the progression of IMT and TPA after a 13-year follow-up; in this study, BMI at baseline did not predict progression of either [8]. Similarly, van der Meer et al. showed that BMI was not associated with an increase in plaque numbers over an average follow-up of 6.5 years [9]. Molino-Lova et al. showed that overweight/obesity, according to BMI category, was not associated with the new formation of plaque in 486 elderly participants without plaque at baseline over a three-year follow-up period [10]. All of these findings agree with the relatively weak association between BMI and the progression of atherosclerosis in our analysis.

On the other hand, not much has been done to clarify the influence of abdominal obesity on the progression of carotid atherosclerosis. One prospective study (*n* = 1894) with a four-year follow-up showed that an increase in WC was one of the determinants of the new formation of plaque among 462 participants without plaque at baseline after adjustment for age, sex, and follow-up time, while BMI was not [11]. However, neither was a determinant of the progression of TPA. In the Rotterdam Study, the determinants of the progression of the number of plaques were analysed in 3409 participants after the 6.5-year follow-up. An increase in WHR was associated with an increase in the number of plaques after adjustment for traditional CVD risk factors. Again, BMI was not associated with increases in the number of plaques [9]. Although a direct comparison of their findings with ours is difficult due to differences in statistical methods and adjustments, the potentially stronger effect of abdominal obesity compared to general obesity is consistent with our results.

The objectives of the studies mentioned above were to investigate the determinants of the progression of atherosclerosis [8, 9, 11] or the association between main exposures other than obesity and atherosclerosis [10]. When restricted to studies directly investigating the association between obesity and atherosclerosis, there is some evidence that IMT in adulthood may partly reflect childhood obesity [19–24]. However, the question of whether or not the development or progression of carotid plaque in adult life is related to obesity in early life has not been investigated.

Most previous studies have used BMI to assess obesity. However, BMI does not provide information about fat distribution. Abdominal obesity, reflecting excess visceral adipose tissue, has a stronger association with inbsulin resistance and dyslipidemia than general obesity [12]. Regarding whether abdominal obesity is more strongly associated with CVD than general obesity, evidence from observational studies is inconsistent. The Emerging Risk Factors Collaboration analysed 221,934 individuals from 58 cohorts and found that BMI, WC, and WHR were all associated with CVD risk, and the authors concluded that their effect sizes were similar [25].

On the other hand, some studies suggest a stronger effect of abdominal obesity than general obesity. The INTERHEART Study, a large multi-centre case-control study with 12,461 myocardial infarction cases, suggested that increased WHR was more strongly associated with the occurrence of myocardial infarction than BMI [26]. Furthermore, the recent INTERSTROKE Study showed that WHR had a stronger association with stroke than BMI had [27].

Recently, two large Mendelian randomisation studies have shed light on the effect of abdominal obesity on CVD [28, 29]. Dale et al. analysed data from 14 prospective studies with 66,842 coronary heart disease cases and 12,389 ischemic stroke cases, and compared associations of genetic risk scores for BMI and WHR adjusted for BMI with various cardiometabolic risks. The results showed that WHR might have a stronger effect on coronary heart disease and stroke than BMI. In particular, only WHR was associated with increased risk of ischemic stroke [29]. Another study, with 111,986 participants from the UK Biobank, showed that a genetic disposition to higher WHR adjusted for BMI was associated with type 2 diabetes and coronary heart disease, supporting causal relationships [28]. These findings emphasise the important role of abdominal obesity. Using BMI may lead to underestimation of the true risk of obesity for CVD.

In the present study, associations were more statistically significant in the analyses using TPA as an outcome than those using the number of plaques. It is expected that continuous plaque variables such as TPA and total plaque volume (TPV) can capture the small change in plaque over time more easily than a simple categorical plaque variable, requiring smaller sample sizes and potentially shorter follow-up time to detect significant changes. It has also been suggested that TPA is likely to be more sensitive to the progression of atherosclerosis than the more commonly studied outcome of IMT because plaque grows along the axis of the artery 2.4 times faster than it changes in thickness [30]. One study, with 349 atherosclerotic patients, compared the predictive ability of future CVD events among TPV, TPA, and IMT after a five-year follow-up [31]. Progression of TPV was significantly associated with CVD events after the adjustment for traditional CVD risk factors. Although the predictive ability of TPA was inferior to TPV, TPA performed better than IMT. While TPV is more sensitive to the progression with its three-dimensional information, TPA would be sensitive enough to detect the progression of plaque burden within a reasonable time frame, which makes TPA an attractive outcome in large population-based studies.

New plaque formation was not associated with any adiposity measures. Considering that our sample was in late adulthood, having no plaque at baseline might mean that participants are to some extent resistant to atherosclerotic changes for genetic or other reasons. This might contribute to the slower progression of carotid atherosclerosis and make it difficult to detect changes in this population. Another potential explanation is lack of power: by restricting the analysis to participants without plaque at baseline, the sample size was almost halved. Besides, like the number of plaques variable, the binary plaque variable provides less statistical information on the progression of plaque burden than a continuous plaque variable capturing size such as TPA.

All effect sizes of observed associations between obesity and the progression of plaque burden in the main analysis were substantially reduced after the adjustment for cardiometabolic risk factors in model 3, and no associations remained significant. This finding is supported by previous studies where strong determinants of the progression of carotid plaque burden included systolic blood pressure and total cholesterol [8, 9]. Our finding validates that the pathway from obesity to carotid atherosclerosis is at least in part through cardiometabolic risk factors.

Recent studies with CVD mortality as an outcome also showed that the risk of obesity for atherosclerotic CVD is largely or fully mediated by these cardiometabolic risk factors [32, 33]. These findings suggest that the strict control of metabolic risk factors might in part attenuate the risk of obesity on the progression of atherosclerosis. Interventions to bring long-term and sustained weight loss through lifestyle change have not been uniformly successful [34, 35]. On the other hand, the effective treatment of cardiometabolic risk factors is established, and with strict control of this might reduce the indirect effect of obesity on atherosclerosis. Nevertheless, the treatment of obesity is vital to block any direct pathway to the progression of atherosclerosis and to control cardiometabolic risk factors better [36]. Because our main objective was to investigate the association between obesity and plaque burden with and without the adjustment for mediators as a whole, this did not require partitioning of the contribution of a specific mediator to the overall effect.

Our major strength is a large sample size with a relatively long follow-up period, which allowed us a reasonable estimation of the association between the progression of carotid atherosclerosis and obesity. Furthermore, theTromsø Study is one of the few prospective studies with repeated carotid plaque measurements. The use of the quantitative plaque variable TPA is another advantage.

Our main limitation is a loss to follow-up. This might reduce the power of the study to detect associations between adiposity and the progression of carotid plaque burden. In addition, selection bias may be introduced. Thus, the association should be interpreted with caution, as should generalisability to the whole population. Another limitation is the possibility of residual confounding. For example, we did not include statin use in the model because statin use was not common at baseline. With respect to smoking, we only had data on this as a binary variable (current smoker: yes/no), which may have resulted in some residual confounding, as it is known that former smokers have a higher CVD risk than non-smokers [37].

Furthermore, we did not consider subsequent changes in CVD risk factors and medications. Moreover, the abdominal obesity index that we used is a crude measure of abdominal adipose tissue. However, it is easily available in a real-world clinical setting. Further study using the reliable measurement of visceral adipose tissue and body composition overall would be informative. Several studies have shown that a decrease in lean mass or skeletal muscle mass was associated with CVD and atherosclerosis [38–40]. In terms of WC, we measured WC at the level of the umbilicus, which tends to give higher WC values compared to other WC measurement sites [41]. However, a systematic literature review concluded that the WC measurement site had little impact on the association of WC with CVD mortality, CVD events, or risk of diabetes [42]. Finally, only the right carotid artery was assessed.

## Conclusion

Abdominal obesity, especially WHtR and WHR, seems to be more strongly associated with the progression of carotid atherosclerosis than general obesity. These associations are largely mediated by established cardiometabolic risk factors.

## Supplementary information

**Additional file 1.**

## Data Availability

The datasets analysed during the current study are available upon the application to the Department of Community Medicine University of Tromsø, [https://en.uit.no/forskning/forskningsgrupper/sub?p_document_id=453582&sub_id=71247]. The application for data access to Tromsø Study 4-6th surveys was requested and approved by the Data and Publication Committee at the University of Tromsø. The Tromsø Study is approved by the Regional Committee for Medical Research Ethics (REK) and the Norwegian Data Inspectorate, and the present study is covered by this approval. Authors received anonymised dataset.

## Abbreviations

BMI: Body mass index
CI: Confidence interval
CVD: Cardiovascular disease
HDL-C: High-density lipoprotein cholesterol
IMT: Intima-media thickness
LDL-C: Low-density lipoprotein cholesterol
SD: Standard deviation
TPA: Total plaque area
WC: Waist circumference
WHR: Waist-to-hip ratio
WHtR: Waist-to-height ratio
