# Supplementary material for: The association between anthropometric measures of adiposity and the progression of carotid atherosclerosis
Source: BMC Cardiovasc Disord. 2020 Mar 17;20:138. doi: 10.1186/s12872-020-01417-0 (PMC7079386; doi:10.1186/s12872-020-01417-0)
Supplement: Supplementary file 1 — Additional file 1. [file 12872_2020_1417_MOESM1_ESM.docx]

**Title**: The association between anthropometric measures of adiposity and the progression of carotid atherosclerosis

**Authors names**

Yume Imahori ^a^ , Ellisiv B. Mathiesen ^b^ , Katy E. Morgan ^c^ , Chris Frost ^c^ , Alun D. Hughes ^d^, Laila A. Hopstock ^e^ , Stein Harald Johnsen ^b^ , Nina Emaus ^f^ , David A. Leon ^a,e^

**Departments and institutions**

^a^Department of Non-communicable Disease Epidemiology, Faculty of Epidemiology and Population Health, London School of Hygiene & Tropical Medicine,WC1E 7HT, UK;

^b^Department of Clinical Medicine, UiT The Arctic University of Norway, Tromsø and Department of Neurology, University Hospital of North Norway, 9037, Tromsø; Norway;

^c^ Department of Medical Statistics, London School of Hygiene & Tropical Medicine, WC1E 7HT, UK;

^d^ Department of Population Science & Experimental Medicine, & MRC Unit for Lifelong Health and Ageing, University College London, WC1E 6BT, UK;

^e^ Department of Community Medicine, Faculty of Health Sciences, UiT The Arctic University of Norway, Tromsø, 9037, Norway;

^f^ Department of Health and Care Sciences, Faculty of Health Sciences, UiT The Arctic University of Norway, Tromsø,9037, Norway

**Contact info:**

Yume Imahori, MD, PhD

Department of Non-communicable Disease Epidemiology, Faculty of Epidemiology and Population Health, London School of Hygiene & Tropical Medicine, Keppel Street, London WC1E 7HT, UK

Tel: +44(0)7597647944 Email: yumeim0405@gmail.com

**Table S1: Participant characteristics at the baseline 4^th^ survey (1994-95) in those scanned at the 5^th^ survey (2001) vs those not scanned at the 5^th^ survey**

| Continuous variable at the 4^th^ survey | The 5^th^ survey carotid scanned | The 5^th^ survey carotid missing | Adjusted for age, sex  β (95%CI) | p-value |
| --- | --- | --- | --- | --- |
| Number | 4829 | 1748 |  |  |
| Age (median) (year) | 60 (55-66) | 65 (58-70) | -0.49 (-0.59, -0.39) | <0.001 |
| Height (cm) | 168.4 (9.4) | 167.9 (9.6) | 0.54 (0.19, 0.89) | 0.003 |
| Weight (kg) | 74.0 (13.1) | 73.6 (14.5) | 0.41 (-0.25, 1.06) | 0.23 |
| BMI (kg/m^2^) | 26.0 (3.8) | 26.0 (4.5) | -0.02 (-0.24, 0.19) | 0.83 |
| WC (cm) | 89.8 (11.1) | 91.1 (12.3) | -0.56 (-1.12, -0.01) | <0.05 |
| WHR | 0.87 (0.08) | 0.88 (0.09) | -0.008 (-0.012, -0.005) | <0.001 |
| WHtR | 0.53 (0.06) | 0.54 (0.07) | -0.005 (-0.009, -0.002) | 0.002 |
| SBP (mmHg) | 143.7 (21.6) | 149.5 (24.5) | -2.79 (-3.93, -1.65) | <0.001 |
| Total cholesterol (mmol/l) | 6.70 (1.27) | 6.72 (1.38) | -0.03 (-0.09, 0.04) | 0.43 |
| Triglycerides (mmol/l) | 1.52 (0.89) | 1.65 (1.06) | -0.13 (-0.18, -0.07) | <0.001 |
| HDL cholesterol (mmol/l) | 1.52 (0.44) | 1.49 (0.46) | 0.03 (0.00, 0.05) | 0.03 |
| LDL cholesterol (mmol/l) | 4.48 (1.17) | 4.49 (1.27) | 0.00 (-0.06, 0.06) | 0.98 |
| HbA1c (%) | 5.4 (0.6) | 5.6 (0.8) | -0.08 (-0.12, -0.04) | <0.001 |
| TPA (median) | 0 (0-13.1) | 7.9 (0-22.9) | -4.01 (-4.93, -3.09) | <0.001 |
| Categorical variable at the 4^th^ survey |  |  |  |  |
| Female (%) | 2490 (51.6%) | 829 (47.4%) | 0.79 (0.71, 0.89) | <0.001 |
| Plaque (1+ vs 0) | 2229 (46.2%) | 1031 (59.0%) | 0.69 (0.61, 0.77) | <0.001 |
| Current Smokers (vs non-smokers) | 1456 (30.2%) | 703 (40.2%) | 0.57 (0.51, 0.64) | <0.001 |
| *Medical history* |  |  |  |  |
| Myocardial infarction | 220 (4.6%) | 187 (10.8%) | 0.48 (0.39, 0.60) | <0.001 |
| Angina pectoris | 377 (7.8%) | 237 (13.6%) | 0.68 (0.57, 0.82) | <0.001 |
| Stroke | 100 (2.1%) | 81 (4.7%) | 0.56 (0.41, 0.76) | <0.001 |
| Diabetes | 114 (2.4%) | 95 (5.5%) | 0.52 (0.39, 0.69) | <0.001 |

BMI: body mass index, CI: confidence interval, HbA1c: glycated haemoglobin, HDL: high-density lipoprotein, LDL: low-density lipoprotein, OR: odds ratio, SBP: systolic blood pressure, TPA: total plaque area, WC: waist circumference, WHR: waist-to-hip ratio, WHtR: waist-to-height ratio, OR: odds ratio,

**Table S2: Participant characteristics at the baseline 4^th^ survey (1994-95): scanned at the 6^th^ survey (2007-08) vs those who scanned at the 6^th^ survey**

| Variable at the 4^th^ survey | The 6^th^ survey carotid scanned | The 6^th^ survey carotid missing | Adjusted for age, sex  β (95%CI) | p-value |
| --- | --- | --- | --- | --- |
| Number | 2974 | 3714 |  |  |
| Age (median) (year) | 58 (53-63) | 65 (59-70) | -1.30 (-1.39, -1.21) | <0.001 |
| Height (cm) | 168.9 (9.3) | 167.7 (9.6) | 0.48 (0.14, 0.82) | 0.005 |
| Weight (kg) | 74.1 (12.7) | 73.7 (14.1) | 0.05 (-0.58, 0.67) | 0.89 |
| BMI (kg/m^2^) | 25.9 (3.5) | 26.2 (4.3) | -0.13 (-0.34, 0.08) | 0.22 |
| WC (cm) | 89.0 (10.7) | 91.1 (12.0) | -0.97 (-1.50, -0.44) | <0.001 |
| WHR | 0.86 (0.08) | 0.88 (0.08) | -0.009 (-0.013, -0.006) | <0.001 |
| WHtR | 0.53 (0.06) | 0.54 (0.07) | -0.007 (-0.011, -0.004) | <0.001 |
| SBP (mmHg) | 139.6 (20.0) | 149.6 (23.4) | -4.23 (-5.32, -3.13) | <0.001 |
| Total cholesterol (mmol/l) | 6.58 (1.24) | 6.81 (1.33) | -0.10 (-0.16, -0.03) | 0.003 |
| Triglycerides (mmol/l) | 1.49 (0.86) | 1.61 (1.00) | -0.11 (-0.16, -0.06) | <0.001 |
| HDL cholesterol (mmol/l) | 1.51 (0.43) | 1.51 (0.45) | 0.02 (-0.01, 0.04) | 0.14 |
| LDL cholesterol (mmol/l) | 4.39 (1.15) | 4.56 (1.22) | -0.07 (-0.13, -0.01) | 0.02 |
| HbA1c (%) | 5.4 (0.4) | 5.6 (0.8) | -0.10 (-0.14, -0.06) | <0.001 |
| TPA (median) | 0 (0-9.6) | 7.2 (0-20.5) | -3.62 (-4.50, -2.73) | <0.001 |
| Categorical variable at the 4^th^ survey |  |  |  |  |
| Female (%) | 1532 (51.5%) | 1849 (49.8%) | 0.80 (0.72, 0.89) | <0.001 |
| Plaque (1+ vs 0) | 1149 (38.6%) | 2172 (58.5%) | 0.67 (0.60, 0.75) | <0.001 |
| Smokers | 833 (28.0%) | 1361 (36.7%) | 0.48 (0.43, 0.54) | <0.001 |
| *Medical history* |  |  |  |  |
| Myocardial infarction | 99 (3.3%) | 315 (8.5%) | 0.54 (0.42, 0.69) | <0.001 |
| Angina pectris | 153 (5.2%) | 470 (12.7%) | 0.59 (0.48, 0.72) | <0.001 |
| Stroke | 30 (1.0%) | 153 (4.1%) | 0.34 (0.22, 0.51) | <0.001 |
| Diabetes | 43 (1.5%) | 170 (4.6%) | 0.45 (0.31, 0.65) | <0.001 |

BMI: body mass index, CI: confidence interval, HbA1c: glycated haemoglobin, HDL: high-density lipoprotein, LDL: low-density lipoprotein, OR: odds ratio, SBP: systolic blood pressure, TPA: total plaque area, WC: waist circumference, WHR: waist-to-hip ratio, WHtR: waist-to-height ratio, OR: odds ratio

**Table S3. Odds ratios of having plaques at the 6^th^ survey (2007-08) among participants without plaques at the baseline 4^th^ survey (1994-95), by adiposity at the 4^th^ survey (incident plaque when plaque is absent at the 4^th^ survey) (n=1639)**

|  | Model 1 OR (95%CI) | p-value | Model 2 OR (95%CI) | p-value | Model 3 OR (95%CI) | p-value |
| --- | --- | --- | --- | --- | --- | --- |
| st BMI | 1.05 (0.93, 1.18) | 0.43 | 1.09 (0.96, 1.23) | 0.18 | 0.99 (0.86, 1.13) | 0.88 |
| st WC | 1.06 (0.94, 1.20) | 0.31 | 1.08 (0.96, 1.22) | 0.19 | 1.00 (0.88, 1.15) | 0.95 |
| st WHR | 1.08 (0.97, 1.21) | 0.17 | 1.08 (0.96, 1.21) | 0.18 | 1.03 (0.91, 1.16) | 0.67 |
| st WHtR | 1.10 (0.97, 1.24) | 0.13 | 1.12 (0.99, 1.27) | 0.08 | 1.02 (0.89, 1.17) | 0.82 |

BMI: body mass index, WC: waist circumference, WHR: waist-to-hip ratio, WHtR: waist-to-height ratio, OR: odds ratio, 95% CI: 95% confidence interval, SD: standard deviation, Model 1: adjusted for age and sex, Model 2: adjusted for variables in Model 1 plus other confounders (smoking, physical activity and education), Model 3: adjusted for variables in Model 2 and mediators (systolic blood pressure, HDL cholesterol, non-HDL cholesterol, glycated haemoglobin, diabetes, lipid and blood pressure-lowering drugs)

**Table S4. The association between the change in the number of plaques between the baseline 4^th^ survey (1994-95) and the 6^th^ survey (2007-08)and a 1 SD increase in baseline adiposity (n=2685)**

|  | Model 1 β (95%CI) | p-value | Model 2 β (95%CI) | p-value | Model 3 β (95%CI) | p-value |
| --- | --- | --- | --- | --- | --- | --- |
| st BMI | 0.005 (-0.036, 0.047) | 0.81 | 0.019 (-0.023, 0.061) | 0.39 | 0.014 (-0.031, 0.059) | 0.55 |
| st WC | 0.004 (-0.038, 0.045) | 0.86 | 0.012 (-0.030, 0.054) | 0.59 | 0.008 (-0.037, 0.053) | 0.74 |
| st WHR | 0.008 (-0.032, 0.048) | 0.70 | 0.009 (-0.032, 0.049) | 0.67 | 0.007 (-0.036, 0.049) | 0.76 |
| st WHtR | 0.012 (-0.030, 0.055) | 0.58 | 0.020 (-0.023, 0.063) | 0.37 | 0.016 (-0.031, 0.062) | 0.51 |

BMI: body mass index, WC: waist circumference, WHR: waist-to-hip ratio, WHtR: waist-to-height ratio, OR: odds ratio, 95% CI: 95% confidence interval, SD: standard deviation, Model 1: adjusted for age and sex, Model 2: adjusted for variables in Model 1 plus other confounders (smoking, physical activity and education), Model 3: adjusted for variables in Model 2 and mediators (systolic blood pressure, HDL cholesterol, non-HDL cholesterol, glycated haemoglobin, diabetes, lipid and blood pressure-lowering drugs)

**Table S5. The association between change in total plaque area (mm^2^) between the baseline 4^th^ survey (1994-95) and the 6^th^ survey (2007-08) and a 1 SD increase in baseline adiposity (n=1515)**

In the 6^th^ survey, participants without plaque are categorised as TPA missing data. Consequently, this analysis includes a smaller number of participants than that using change in the number of plaques between the 4^th^ survey and the 6^th^ survey

|  | Model 1 β (95%CI) | p-value | Model 2 β (95%CI) | p-value | Model 3 β (95%CI) | p-value |
| --- | --- | --- | --- | --- | --- | --- |
| st BMI | -1.009 (-2.172, 0.155) | 0.09 | -0.694 (-1.877, 0.489) | 0.25 | -0.971 (-2.233, 0.290) | 0.13 |
| st WC | -0.479 (-1.662, 0.704) | 0.43 | -0.269 (-1.464, 0.926) | 0.66 | -0.529 (-1.802, 0.743) | 0.42 |
| st WHR | -0.168 (-1.278, 0.943) | 0.77 | -0.122 (-1.235, 0.990) | 0.83 | -0.294 (-1.448, 0.859) | 0.62 |
| st WHtR | -0.801 (-2.010, 0.408) | 0.19 | -0.593 (-1.819, 0.633) | 0.34 | -0.895 (-2.193, 0.402) | 0.18 |

BMI: body mass index, WC: waist circumference, WHR: waist-to-hip ratio, WHtR: waist-to-height ratio, OR: odds ratio, 95% CI: 95% confidence interval, SD: standard deviation, Model 1: adjusted for age and sex, Model 2: adjusted for variables in Model 1 plus other confounders (smoking, physical activity and education), Model 3: adjusted for variables in Model 2 and mediators (systolic blood pressure, HDL cholesterol, non-HDL cholesterol, glycated haemoglobin, diabetes, lipid and blood pressure-lowering drugs)

**Appendix**

Due to concerns over normality assumptions, bias-corrected and accelerated bootstrap confidence intervals (CI) were estimated from 10,000 bootstrap resamples for all linear regression models. However, the differences between these and standard CIs tended to be relatively small, we present standard CIs in the results section.
